# Report of a Joint Committee of the Phila. Co. Med. Society, &c.

**Published:** 1854-02

**Authors:** 


### Rrport of a Joint Committee of the Philadelphia County Medical Society and the Philadelphia College of Pharmacy, relative to Physicians' Prescriptions.

[*Published by order of the Board of Trustees of the Philadelphia Coll. of Pharm.*]

The joint Committees of the Philadelphia County Medical Society, and of the Philadelphia College of Pharmacy, appointed for the purpose of considering the means best adapted to prevent the occurrence of mistakes in the compounding of the prescriptions of Physicians by Apothecaries, beg leave to report that they have given to the subject all the attention that its importance demands, and present the following hints as the results of their joint deliberations. They have taken the liberty of adding, also, a few general hints on the relations that should exist between physicians and pharmaceutists.

### A. *In Respect to Physicians.*

1. Physicians should write their prescriptions carefully and legibly, making use of good paper, and, whenever possible, of pen and ink. When obliged to write with a pencil, they should take the precaution to fold the prescription twice, so as to prevent its being defaced.

2. The nomenclature of the United States Pharmacopœia is becoming annually more in favor with pharmaceutists; a statement attested by the fact that 1500 copies of the book of Latin Labels for shop furniture, published by the Philadelphia College of Pharmacy, have been disposed of within three years. Physicians are also becoming more alive to the merits of our national Codex, and they are respectfully urged to familiarize themselves with its nomenclature, and to adhere to it strictly in their prescriptions.

3. The numerous treatises on Materia Medica, Pharmacy and the Practice of Medicine, of English origin, that are reprinted in this country, notwithstanding they are generally interlarded with the formulæ of our own Pharmacopœia, tend, nevertheless, very much to confuse the physician and apothecary, in the use and exact meaning of terms in prescriptions. To obviate the difficulties thus occasioned, the physician should, when he prescribes a medicine, which is not officinal, nor in common use, state on his prescription, either in a note at the bottom, or within parenthesis, following the article, the authority or work from whence it is derived, as "Griffith's Formulary,"—"Ellis' Formulary,"—"Braithwaite's Retrospect," etc.

4. Physicians would lesson the risk of errors in their prescriptions, and increase the chances of their detection should they be made, by observing the following hints.

1st. Write the name of the patient at the top of the prescription, unless a good reason prevents this being done, in which case, it should be expressed as for Mr. G—, Mrs. R—, or Mrs. S.'s child, or for Master T—, so as to convey to the apothecary some idea of the age of the patient.

2d. The date and name of the physician or his initials, should always be appended, and, whenever practical, the dose and mode of administering the medicine directed.

3d. When an unusually large dose of an active medicine is prescribed, as opium, morphia, elaterium, strychnia, etc., let such names be put in *italics*, and the quantity or quantities repeated in writing enclosed within a parenthesis; thus:—R Morphiæ Sulphatis grs. vj. (six grains.) Div. in chart. vj.

4th. When an active substance is to be used externally, it should be so stated on the prescription; thus, "For external application" "To be applied to the part as directed," etc.

5th. The quantities of each article should be placed in a line with the name, and not below it, and in using the Roman numerals, the *i*'s should be dotted correctly.

6th. The occasional practice of writing the directions intended for the patient in *latin*, and especially in abbreviated latin, is uncalled for, and attended with some risk; it is far safer to write them in English, and without abbreviation or the use of figures, unless these are well and distinctly formed.

B. *In Respect to the Apothecary.*

1st. The apothecary should hesitate to dispense a prescription, the handwriting of which is so imperfect as to render the writer's meaning doubtful—especially if it involves agents of a poisonous or irritating character—unless he is able, from collateral circumstances, to satisfy himself of the intent of the prescriber. In such a case he should delay the delivery of the medicine to the patient until he can see the physician, and in doing so he should avoid committing the latter, by agreeing to send the medicine when it is ready.

2d. The apothecary is justified in the same means of delay, if he, after deliberate consideration, believes that the physician has inadvertently made a mistake in the quantity or dose of the article or articles prescribed; always keeping in view the physician's reputation as well as his own. Every respectful application, in such cases, to a physician, should be met in good faith and with kind feeling, even though no error should prove to exist.

3d. In his demeanor and language, the apothecary should cautiously avoid compromising the physician, unless it be unavoidable, in which case honesty is the best policy, and the patient or his messenger should be told that it will be necessary to have an interview with the physician previously to compounding his prescription.

4th. The apothecary is not justifiable in making inquiries relative to the patient or his disease, or remarks relative to the character or properties of the medicines prescribed, that are uncalled for, or likely to convey a wrong impression, through an ignorant messenger, to the patient, excepting it to be done in a case where he has doubts in regard to the prescription, and wishes to satisfy himself, and where he should act with great discreetness.

5th. When an apothecary is asked his opinion of a physician's prescription in a manner that indicates want of faith in the prescriber, he should waive the question, unless by a direct answer he should be able to restore the confidence. When asked the nature of the ingredients, he should be guided in his answer by circumstances, avoiding to give the desired information, when he believes it would be contrary to the wish of the physician, or attended with injurious consequences. In other cases he should use his own judgment.

6th. Physicians being often unacquainted with practical pharmacy, pay little attention to the order in which the several articles entering into a prescription are arranged, with the view to facilitate the operations of dispensing. It hence becomes the first duty of the apothecary carefully to read the prescription and fix the proper order in his mind. He should, at the same time, acquire the habit of considering the quantities ordered in relation to the usual doses, and, also, the general bearing of the prescription; and a constant resort to this practice, based on due knowledge, must almost inevitably detect mistakes, if any have been made.

7th. Apothecaries should accustom their assistants to study prescriptions in this light, and to acquire such a knowledge of the doses and therapeutical uses of medicines as shall serve to guide them in avoiding errors.

8th. The apothecary, when engaged in dispensing a prescription, should, as far as possible, avoid mental pre-occupation, and give his attention fully to his task. He should acquire the habit of *always*

examining the label of the bottle before using its contents, and he should satisfy himself that he has read the prescribed quantity correctly, by referring to the prescription anew before weighing out each article. It is also, a useful precaution to have bottles containing mineral or vegetable poisons, distinguished by some prominent mark.

9th. As the conscientious discharge of his duty should be the aim of every apothecary, seeing that on his correct action depends, in no slight degree, the usefulness of the physician, no pain should be spared to secure the efficiency of the medicines dispensed, whether they be drugs or preparations. The latter should always be prepared of full strength, and according to the formulæ recognized by the United States Pharmacopœia, unless when otherwise specially ordered.

10th. The apothecary should always label, and number correctly, all medicine dispensed by him on the prescription of a physician ; he should, also, invariably, transcribe on the label, in a plain legible hand writing, the name of the patient, the date of the prescription, the directions intended for the patient, and the name or the initials of the prescriber.

11th. The original prescription should always be retained by the apothecary, whose warantee it is, in case of error on the part of the prescriber. When a copy is requested, if, as in many instances, no objection can be urged, it should be a *fac simile* in language and symbols, and not a translation.

12th. In no instance is an apothecary justifiable in leaving his business in charge of boys, or incompetent assistants—or in allowing such to compound prescriptions, excepting under his immediate and careful supervision.

13th. In justice to his sense of the proper limits of his vocation, to the medical profession, and to his customers, the apothecary should abstain from prescribing for diseases, excepting in those emergencies, which occasionally occur, demanding immediate action, or, in those every day unimportant cases where to refuse council would be construed as a confession of ignorance, calculated to injure the reputation of the apothecary, and would be attended with no advantage to either physician or patient.

14th. The sale of quack or secret medicines, properly so called, constitutes a considerable item in the business of some apothecaries. Many of the people are favorably impressed towards that class of medicines, and naturally go to their apothecaries for them. It is this which has caused many apothecaries to keep certain of these nostrums, who are ready and willing to relinquish the traffic in them, but for the offence that a refusal to supply them to their customers would create. At present all that the best disposed apothecary can be expected to do, is to refrain from the manufacture himself of quack and secret medicines; to abstain from recommending them, either verbally or by exhibiting show bills, announcing them for sale, in his shop or windows; and to discourage their use, when appealed to.

15th. Having in view the welfare of the community and the advancement of pharmaceutic science and interest, it is all important that the offices of prescribing and compounding medicines should be kept distinct, in this city and surrounding districts. All connection with, or moneyed interest in apothecary stores, on the part of physicians, should, therefore, be discountenanced. With respect to the pecuniary understanding said to exist, in some instances, between apothecaries and physicians, we hold, that no well disposed apothecary or physician would be a party to such contract, and consider the code of Ethics of the College of Pharmacy and the Constitution of the Philadelphia County Medical Society as sufficiently explicit on this subject.

16th. In reference to the patronage on the part of Physicians of particular apothecaries, we are of opinion, as a general rule, that Graduates in Pharmacy should be encouraged in preference to others of the same date of business, and whilst admitting the abstract right of the physician to send his prescription where he pleases, we think that justice should dictate the propriety of his encouraging the nearest apothecary deserving of his confidence and that of the patient.

| | |
|---|---|
| D. Francis Condie,<br>Wm. Maybury,<br>G. Emerson. | *Committee of County Medical Society.* |
| William Procter, Jr.,<br>H. C. Blair,<br>John H. Ecky. | *Committee of Philadel. College of Pharmacy.* |

---

We publish the above from the *American Journal of Pharmacy*, because we think it defines clearly and in the main correctly the duties of apothecaries and physicians. In our cities and larger towns, the office of prescribing and dispensing, should as far as possible be kept separate. This arrangement, allowing as it does, the physician to select from the materia medica irrespective of what he may happen to have in his medicine case, those agents best adapted to meet the therapeutical indications, and securing in the preparation and dispensing of those agents that care, which undivided attention and constant experience brings, promises for the patient a greater degree of safety, and for the practitioner a more satisfactory success than could be obtained by any other course.

We think it can rarely become necessary for the apothecary to tell the messenger that an interview with the physician must be had before the prescription can be confounded. (B. art. 3.) We cannot admit that the apothecary should ever make inquiries relative to the nature of the disease, even though he has doubts in regard to the prescription. (B. art. 4.) In such cases the physician only should be consulted. When an apothecary is asked the nature of the ingredients of a prescription, he should *always* in our opinion refer the patient to the physician. (B. art. 5.) With these reservations, and they are trifling in their character, we give our unqualified assent to to the rules laid down for the government of these two branches of the profession. They are at least applicable to Chicago. J

